# BRAF V600E mutations are characteristic for papillary craniopharyngioma and may coexist with CTNNB1-mutated adamantinomatous craniopharyngioma

**DOI:** 10.1007/s00401-014-1270-6

**Published:** 2014-04-09

**Authors:** Sarah Jane Larkin, Veronica Preda, Niki Karavitaki, Ashley Grossman, Olaf Ansorge

**Affiliations:** 1 Nuffield Department of Clinical Neurosciences, Department of Neuropathology, John Radcliffe Hospital, Headley Way, Oxford, OX3 9DU UK; 2 Kolling Institute, Royal North Shore Hospital, University of Sydney, Pacific Hwy, St Leonards, NSW 2065 Australia; 3 Department of Endocrinology, Oxford Centre for Diabetes, Endocrinology and Metabolism, Churchill Hospital, Old Rd, Headington, Oxford, OX3 7LE UK

Craniopharyngiomas are epithelial, sellar tumours comprising two subtypes: adamantinomatous (aCP) and papillary (pCP). aCPs contain mutations in CTNNB1, encoding β-catenin: a component of the adherens junction and mediator of Wnt signalling. Reported frequency of CTNNB1 mutations varies widely (16–100 %) [6, 7]. Recently, it was reported that pCPs contain BRAF p.V600E mutations in 95 % of cases [2] and that CTNNB1 and BRAF mutations are mutually exclusive and specific to tumour subtype. We examined the relationship between mutation in CTNNB1 and BRAF and subcellular location of β-catenin in a series of 37 craniopharyngiomas. The region of BRAF exon 15 containing codon 600 was sequenced, as was exon 3 of CTNNB1. Immunohistochemistry (IHC) for β-catenin was used to examine its subcellular location and an antibody specific for the BRAF V600E mutation (clone VE1) was used to complement the sequencing findings in all aCP and pCPs. Methods are reported in Online Resource 1.

We found BRAF V600E mutations in 81 % (17 of 21) pCPs by targeted Sanger sequencing and in 86 % (18 of 21) pCPs by IHC. Although there was agreement between methods in 95 % (20 out of 21) of cases, interpretation of anti-BRAF V600E staining was challenging due to occasional non-specific reactivity. aCP cases were selected for the study on the basis of their CTNNB1 mutation status [6 wild type and 10 mutant (9 T41I, 1 D32N)]. In all aCPs, translocation of β-catenin from membrane to cytosol/nucleus was observed, confirming the utility of β-catenin translocation as a diagnostic tool. Of 16 aCP cases, 14 (88 %) were BRAF wild type by sequencing and IHC. We observed BRAF V600E mutation in two aCP cases. This finding was validated by careful diagnostic review of morphology and comparison with IHC findings. Further validation was obtained by sequencing in forward and reverse directions from two DNA samples extracted on different occasions. In both these specimens, CTNNB1 mutation was also present (T41I) (Fig. 1).

**Fig. 1:**
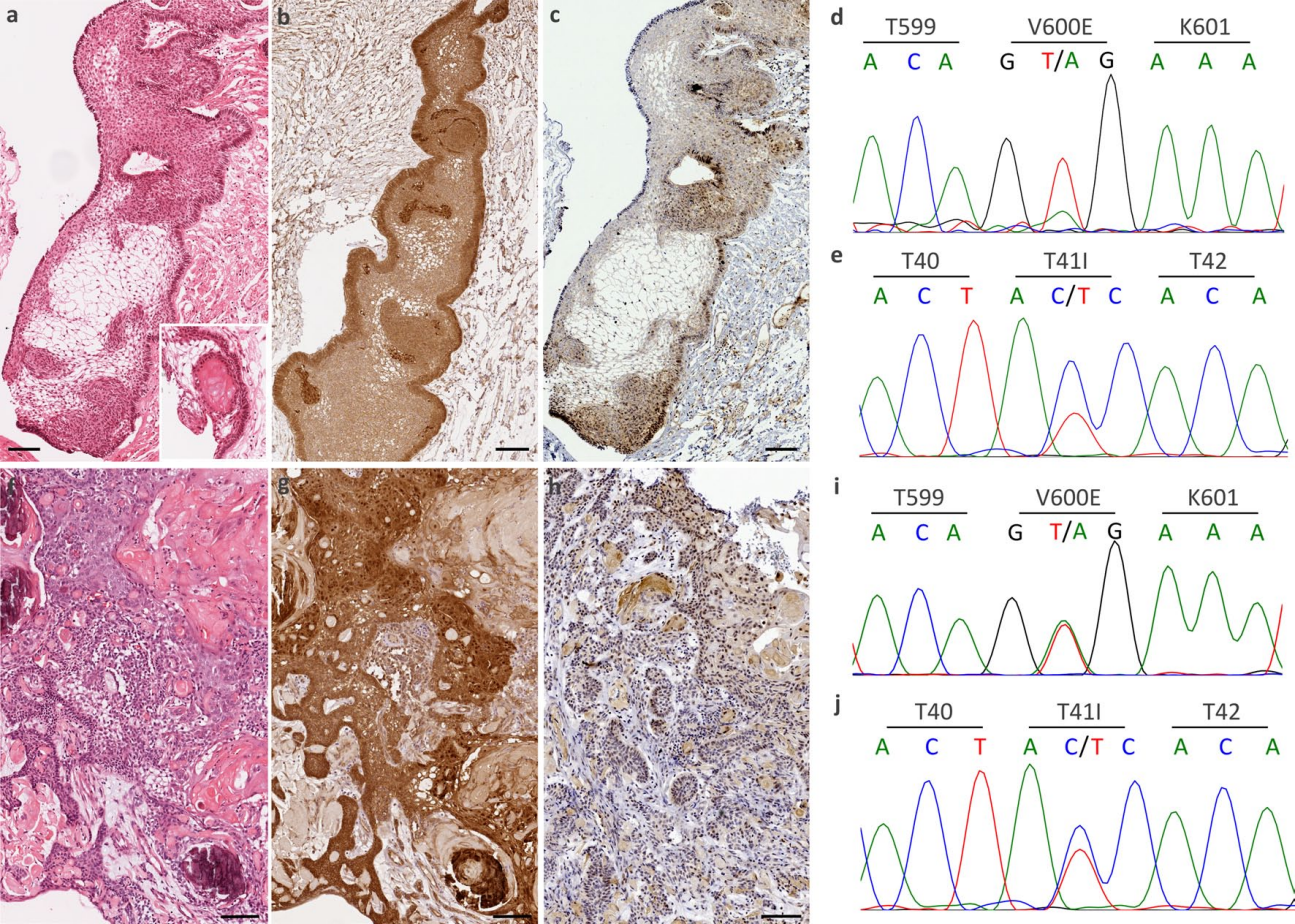
BRAF V600E mutations in adamantinomatous craniopharyngioma. Two cases are shown (**a**–**e** and **f**–**j**). **a**, **f** Classical features of aCP (wet keratin, stellate reticulum, palisaded epithelium). **b**, **g** Translocation of β-catenin is shown in both cases (*brown* reaction product). **c**, **h** An antibody to BRAF V600E (VE1) shows staining of the tumour tissue (*brown* reaction product). **d**, **i** V600E mutations are seen in BRAF. **e**, **j** T41I mutations are seen in CTNNB1. All scale bars 100 μm

Sequencing of bulk tumour revealed no relationship between CTNNB1 mutation and cytosolic/nuclear accumulation of β-catenin. However, our study did not search for mutations at other loci, which could have led to an under-estimation of the proportion of specimens harbouring CTNNB1 mutations. In other studies on FFPE tissue that used Sanger dideoxy sequencing to search for CTNNB1 mutation, the rate was also not 100 % [3, 5, 7], suggesting that this method is not sufficiently sensitive to detect the presence of a mutation in all samples. In contrast, Brastianos et al. [2] found CTNNB1 mutations in 96 % of aCPs using mass spectrometric genotyping.

We found BRAF V600E mutations in 81 % of pCPs. Difficulties interpreting BRAF V600E staining have been reported previously and suggest that in specimens with a significant amount of epithelium, sequencing may be more reliable for determining mutational status of BRAF [1, 8]. A mutation in BRAF was found in two aCP cases, both of which had a coexisting CTNNB1 mutation, demonstrating that although the majority of aCPs do not contain BRAF mutations, they are not exclusive to pCPs and can exist with mutations in CTNNB1. In the majority of cases, however, mutations segregate with tumour subtype: CTNNB1 in aCPs and BRAF in pCPs.

While inhibition of the Wnt pathway has proved challenging and effective inhibitors are still largely in development, inhibitors of mutant BRAF have shown efficacy as chemotherapeutic agents in the treatment of melanoma [4]. The finding that BRAF V600E is mutated in the majority of pCPs offers the possibility for targeted BRAF-inhibitor therapy for patients with this tumour type.

## Electronic supplementary material

Below is the link to the electronic supplementary material.

Supplementary material 1 (PDF 213 kb)

## References

[1] Adackapara CA, Sholl LM, Barletta JA, Hornick JL (2013). Immunohistochemistry using the BRAF V600E mutation-specific monoclonal antibody VE1 is not a useful surrogate for genotyping in colorectal adenocarcinoma. Histopathology.

[2] Brastianos PK, Taylor-Weiner A, Manley PE, Jones RT, Dias-Santagata D, Thorner AR, Lawrence MS, Rodriguez FJ, Bernardo LA, Schubert L, Sunkavalli A, Shillingford N, Calicchio ML, Lidov HG, Taha H, Martinez-Lage M, Santi M, Storm PB, Lee JY, Palmer JN, Adappa ND, Scott RM, Dunn IF, Laws ER, Stewart C, Ligon KL, Hoang MP, Van Hummelen P, Hahn WC, Louis DN, Resnick AC, Kieran MW, Getz G, Santagata S (2014). Exome sequencing identifies BRAF mutations in papillary craniopharyngiomas. Nat Genet.

[3] Buslei R, Nolde M, Hofmann B, Meissner S, Eyupoglu IY, Siebzehnrubl F, Hahnen E, Kreutzer J, Fahlbusch R (2005). Common mutations of beta-catenin in adamantinomatous craniopharyngiomas but not in other tumours originating from the sellar region. Acta Neuropathol.

[4] Flaherty KT, Puzanov I, Kim KB, Ribas A, McArthur GA, Sosman JA, O’Dwyer PJ, Lee RJ, Grippo JF, Nolop K, Chapman PB (2010). Inhibition of mutated, activated BRAF in metastatic melanoma. N Engl J Med.

[5] Kato K, Nakatani Y, Kanno H, Inayama Y, Ijiri R, Nagahara N, Miyake T, Tanaka M, Ito Y, Aida N, Tachibana K, Sekido K, Tanaka Y (2004). Possible linkage between specific histological structures and aberrant reactivation of the Wnt pathway in adamantinomatous craniopharyngioma. J Pathol.

[6] Oikonomou E, Barreto DC, Soares B, De Marco L, Buchfelder M, Adams EF (2005). Beta-catenin mutations in craniopharyngiomas and pituitary adenomas. J Neurooncol.

[7] Sekine S, Shibata T, Kokubu A, Morishita Y, Noguchi M, Nakanishi Y, Sakamoto M, Hirohashi S (2002). Craniopharyngiomas of adamantinomatous type harbor beta-catenin gene mutations. Am J Pathol.

[8] Sperveslage J, Gierke M, Capper D, Honegger J, Sipos B, Beschorner R, Schittenhelm J (2013). VE1 immunohistochemistry in pituitary adenomas is not associated with BRAF V600E mutation. Acta Neuropathol.

