# Supplementary material for: BRAF V600E mutations are characteristic for papillary craniopharyngioma and may coexist with CTNNB1-mutated adamantinomatous craniopharyngioma
Source: Acta Neuropathol. 2014 Apr 9;127(6):927–9. doi: 10.1007/s00401-014-1270-6 (PMC4024131; doi:10.1007/s00401-014-1270-6)
Supplement: Supplementary file 1 — Supplementary material 1 (PDF 213 kb) [file 401_2014_1270_MOESM1_ESM.pdf]

Immunohistochemistry

Immunohistochemical analysis was performed on 4µm sections from FFPE tumour specimens. Following dewaxing through graded alcohols, endogenous peroxidase activity was blocked (3% (v/v) H_2O_2 in PBS, pH 7.3, 20 minutes with orbital shaking). Epitope retrieval was achieved by autoclaving in sodium citrate (10mM, pH 6.0, 10 minutes) (β-catenin) or Tris-EDTA (10mM Tris base, 1mM EDTA, pH 9.0, 10 minutes) (BRAF). Sections were blocked with serum block (HiStar 3000 kit, AbD serotec (Oxford, UK)) for 15 minutes, then incubated overnight at 4°C with primary antibody diluted in PBS or TBS-T (1:1000 in PBS (β-catenin); 1:50 in TBS-T (BRAF)). All subsequent steps were carried out according to manufacturer's instructions (AbD serotec) with the following modifications: HRP polymer was applied for 40 minutes and DAB for 5 minutes. Sections were counterstained with Cole's haematoxylin.

Sequencing of CTNNB1 exon 3 and BRAF exon 15

DNA was extracted from 5 x 10µm sections of FFPE tissue from archival surgical specimens (QiaAmp FFPE DNA kit, Qiagen (Crawley, UK)). PCR was performed to generate a 269-bp amplicon including codons S33, S37, T41 ad S45 in exon 3 of CTNNB1. Primers were: sense (5'-GATTTGATGGAGTTGGACATGG -3') and antisense (5'- TGTTCTTGAGTGAAGGACTGAG -3'). DNA template (50ng) was added to 10 x PCR buffer solution (10% v/v, Qiagen), $MgCl_2$ (final concentration 4mM, Qiagen), dNTPs (200µM) (Promega, Southampton, UK) 0.5U of Taq polymerase (HotStarTaq Plus, Qiagen) and 200nM each of sense and antisense primers in a total volume of 20µl. Cycling conditions were: 95°C for 5 minutes, followed by 35 cycles of 95°C for 30 seconds, 60ºC for 30 seconds and 72°C for 1 minute. To generate a 173-bp amplicon including codon V600 in exon 15 of BRAF, primers were: sense (5' – TGCTTGCTCTGATAGGAAAATG – 3') and antisense (5' – CCACAAAATGGATCCAGACA – 3') [1]. DNA template (100ng) was added to 10 x PCR buffer solution (10% v/v, Qiagen), $MgCl_2$ (final concentration 4mM, Qiagen), dNTPs (200µM) (Promega, Southampton, UK) 0.5U of Taq polymerase (HotStarTaq Plus, Qiagen) and 400nM each of sense and

antisense primers in a total volume of 20µl. Cycling conditions were: 95°C for 5 minutes, followed by 35 cycles of 95°C for 30 seconds, 60ºC for 30 seconds and 72°C for 40sec, and a final elongation step at 72ºC for 1 minute. Products were examined by agarose gel separation and purified (MinElute PCR Purification kit (Qiagen)). Sequencing reactions were performed using BigDye Terminator chemistry and an ABI-3730 sequencer.

1. Capper D, Preusser M, Habel A, Sahm F, Ackermann U, Schindler G, Pusch S, Mechtersheimer G, Zentgraf H, von Deimling A (2011) Assessment of BRAF V600E mutation status by immunohistochemistry with a mutation-specific monoclonal antibody. Acta Neuropathologica 122 (1):11-19. doi:10.1007/s00401-011-0841-z
